# Impact of type of anaesthesia on long-term outcomes in glioma patients undergoing craniotomy: A systematic review

**DOI:** 10.12669/pjms.41.13(PINS-NNOS).13486

**Published:** 2025-12

**Authors:** Aqsa Mughal, Sidra Ameer, Arooj Kiran, Mazhar Nasim, Haseeb Mehmood Qadri, Shahzad Hussain Shah

**Affiliations:** 1 Dr Aqsa Mughal, MBBS. Salford Royal Hospital, Salford, England; 2 Dr. Sidra Ameer, MBBS.King’s College Hospital NHS Trust, London, England; 3 Dr. Arooj Kiran, MBBS. Punjab Institute of Neurosciences, Lahore, Pakistan; 4 Dr. Mazhar Nasim, MBBS. Salford Royal Hospital, Salford, England; 5 Dr. Haseeb Mehmood Qadri, MBBS. Punjab Institute of Neurosciences, Lahore, Pakistan; 6 Dr. Syed Shahzad Hussain Shah, FCPS. Punjab Institute of Neurosciences, Lahore, Pakistan

**Keywords:** Anesthesia, Brain Neoplasms, Glioma, Neurosurgical Procedures, Treatment Outcome

## Abstract

**Objective:**

To assess the effects of volatile and intravenous anaesthetic agents on long-term outcomes in patients with glioma, undergoing tumour resection.

**Methodology:**

This systematic review was performed following Preferred Reporting Items for Systematic Reviews and Meta-Analysis (PRISMA) guidelines. Two databases were searched from inception until January 2025, namely PubMed/MEDLINE and EMBASE using MeSH terms for anaesthesia and glioma. Six retrospective cohort studies were selected for data extraction after thoroughly reviewing the database search results against pre-determined inclusion and exclusion criteria. The quality assessment was done as per National Heart, Lung and Blood institute checklist for observational studies.

**Results:**

A total of six retrospective cohort studies conducted between 2017 and 2021 were reviewed. These included both sexes, predominantly aged over 18 years, except for one study that enrolled patients as young as 16. Five studies concluded that the type of anaesthesia had no effect on survival or recurrence. Only one associated propofol with increased progression free survival (PFS) and overall survival (OS). While rest of the studies showed no effect, Dong et al. suggested that sevoflurane may reduce OS in patients with Karnofsky Performance Status (KPS) <80. Overall, the type of general anaesthesia drugs did not affect five-year PFS rate.

**Conclusion:**

We found no impact of anaesthetic agent on glioma outcomes in majority of studies, but the literature suggesting potential tumour-modulating effects of these drugs is significant enough to warrant further studies. Similar associations exist in other cancers. Well-designed RCTs are crucial to clarify if anaesthetic agents affect long-term survival and oncological outcomes in glioma patients.

## INTRODUCTION

Gliomas are most common primary malignant brain tumours, accounting for 80% of all brain tumours. With high prevalence & poor prognosis, they pose significant challenges in neuro-oncology.^1^ They have an incidence rate estimated to be approximately 3-5 per 100,000 per annum, with glioblastoma multiforme (GBM) being the most common and aggressive subtype.^1^ Surgical intervention remains first choice, with the extent of resection being a key prognostic factor.^2^ Even with total resection of high-grade gliomas, recurrence remains a frequent and challenging issue.^3^ Adjunct treatments of Radiotherapy, chemotherapy with Temozolomide have been shown to improve survival outcomes when used in combination.^4^ Despite current interventions, gliomas remain highly resistant to treatment, for example GBM has median survival rates of 12–16 months for World Health Organization grade III and IV tumours with survival rate of 1–2 years; this has prompted numerous experimental studies at the molecular level to explore the role of stem cell niches and their micro-environment in potentially overcoming treatment resistance.^5^,^6^

Anaesthetic agents have been implicated to trigger immunosuppression, which can influence how residual cells after surgery will behave.^3^ The balance between pro-inflammatory (IL-6, IL-8) and anti-inflammatory cytokines (IL-10) is influenced by anaesthesia and surgery, which in turn leads to increased rate of post-operative complications and cancer progression.^7^ Anaesthetic agent choices include use of local anaesthetic agents along with a continuous infusion protocol during awake craniotomies or general anaesthesia using either inhalational agents (isoflurane, sevoflurane, desflurane) or total intravenous anaesthetic agents (propofol) for tumour resection. Some anaesthetists prefer combining both volatile and intravenous anaesthetics to reduce the adverse effects of each (like nausea and vomiting caused by volatile drugs).^8^

Even though the full impact of general anaesthetics on cellular processes remains unclear, there is increasing evidence that these agents influence gene expression in both animal and human cells. Some experimental studies have shown that volatile anaesthetics modulate gene expression in a time dependent manner.^9^ In one study done by Iwasaki M, these agents have also been found to inhibit natural killer cell activity and increasing hypoxia-inducible factor (HIF) leading to tumour cell proliferation and metastasis.^10^ For example, Sevoflurane treatment (a volatile anaesthetic) demonstrates anti-metastasis role in glioma cells in in-vitro.^11^-^13^ Propofol has also been found to have some anticancer effects, by inhibiting glial cell proliferation, inducing apoptosis and suppressing and invasion through involving NF-kb, PI3K/AKT and MMP pathways.^14^ On contrary, a study done by Fan et.al showed propofol, to cause glioma cell proliferation in animals by enhancing stem like properties of tumour tissue.^13^ Also, a retrospective analysis of around 7000 patients undergoing cancer surgery found 50% higher mortality with volatile anaesthesia compared to total IV anaesthesia.^15^

Essentially, available studies suggest conflicting data for the effects of anaesthetic agents on cancer cell behaviour during excision of glioma. Some of them suggest that these drugs cause proliferation of glial cells like Iwasaki et al.^10^ among others while others suggest they have an anti-inflammatory or suppressive effect on glial cell proliferation like Fan et al.^13^ and Wigmore et al.^15^ There is a spectrum of outcomes but a consensus on these is yet to emerge. This review aims to bridge this gap by critically appraising available resources. The rationale of the study was to provide a cohesive picture on the impact of different anaesthesia types on long-term outcomes and it also highlights the unanswered research questions to guide future studies and promote more comprehensive understanding of the influence of anaesthetic interventions beyond their inherent function of anaesthesia. This study could help formulate perioperative anaesthetic strategies to promote long term outcome.

## METHODOLOGY

This systematic review was conducted according to the guidelines of the Preferred Reporting Items for Systematic Reviews and Meta-Analysis (PRISMA) checklist for the literature with no time restriction. Existing literature published from inception until January 2025 was searched on PubMed/MEDLINE and EMBASE using Boolean operators AND, OR with the following strategy: (Anaesthesia OR anaesthetic agent) AND (Glioma OR Brain tumour) AND (Neurosurgery OR craniotomy). The manual search of references and relevant articles for included studies was performed by two independent reviewers AM & AK.

### Inclusion criteria:

- Retrospective cohort studies focusing on impact of volatile and intravenous anaesthetics on long-term outcomes of population with glioma undergoing tumour resection.
- Studies involving adult patients with glioma.
- Patients underwent tumour resection under general anaesthesia or had awake craniotomy using intravenous, volatile anaesthetic drugs, or combined anaesthesia.

### Exclusion criteria

- Study design other than Cohort Studies (case reports, animal studies, systematic reviews, literature review, study protocols of RCTs)
- Studies discussing the effect of Anaesthetics on cancers other than glioma
- Studies only monitoring Perioperative & early postoperative complications

### Data analysis technique:

Search results from the two databases were imported to Endnote to remove any duplicates. Two reviewers SA & MN performed title and abstract screening (when available) followed by the full-text screening of the included articles by using the eligibility criteria that was already determined. The data was extracted and cross-matched for accuracy by a third reviewer (HMQ). The name of authors, sample size, participant characteristics, inclusion and exclusion criteria, sample size, choice of anaesthesia, time to follow up, adjuvant treatment, time period studies by the article and outcomes were recorded in tables. The studies were compared for their sample sizes and other salient features particularly primary and secondary outcomes. A qualitative analysis was done which is explained in results using the descriptive technique. A comparison of volatile and intravenous anaesthetics on long term outcomes highlighting recurrence, progression free survival and overall survival was made. Two authors, AM & SA, assessed the quality of the studies without blinding to authorship or journal, using quality assessment tools available on National Heart Lung & Blood Institute website for observational studies.^16^ (Table-I) The review found that all studies were adjusted for confounders that could create bias. Also exposure was never reassessed over time and follow up (FU) loss was variably reported.

**Table-I:** Quality Assessment using NHLBI tool for cohort/observational studies.

	Grau et al.^17^		Cata et al.^18^		Dong et al.^19^		Chowdhury et al.^20^		Huang et al.^21^		Schmoch et al.^22^	
	Reviewer 1	Reviewer 2	Reviewer 1	Reviewer 2	Reviewer 1	Reviewer 2	Reviewer 1	Reviewer 2	Reviewer 1	Reviewer 2	Reviewer 1	Reviewer 2
Q1	Yes	Yes	Yes	Yes	Yes	Yes	Yes	Yes	Yes	Yes	Yes	Yes
Q2	Yes	Yes	Yes	Yes	Yes	Yes	Yes	Yes	Yes	Yes	Yes	Yes
Q3	NA	Yes	NA	NA	NA	NA	NA	NA	NA	Yes	NA	No
Q4	Yes	Yes	CD	Yes	Yes	Yes	Yes	Yes	Yes	Yes	Yes	Yes
Q5	No	No	Yes	Yes	Yes	Yes	Yes	Yes	No	Yes	Yes	Yes
Q6	Yes	Yes	Yes	Yes	Yes	Yes	Yes	Yes	Yes	Yes	Yes	Yes
Q7	Yes	Yes	Yes	Yes	Yes	Yes	Yes	Yes	Yes	Yes	Yes	Yes
Q8	NA	NA	NA	Yes	NA	Yes	NA	No	NA	No	Yes	No
Q9	Yes	Yes	Yes	Yes	Yes	Yes	Yes	Yes	Yes	Yes	Yes	Yes
Q10	No	No	No	No	No	No	No	No	No	No	No	No
Q11	Yes	Yes	Yes	Yes	Yes	Yes	Yes	Yes	Yes	Yes	Yes	Yes
Q12	No	No	No	No	Yes	Yes	Yes	Yes	NA	Yes	No	No
Q13	NA	NA	NA	Yes	NA	NA	No	No	Yes	Yes	No	No
Q14	Yes	Yes	Yes	Yes	Yes	Yes	Yes	Yes	Yes	Yes	Yes	Yes

CD: Cannot determine, NA: Not applicable.

## RESULTS

**Fig.1:**
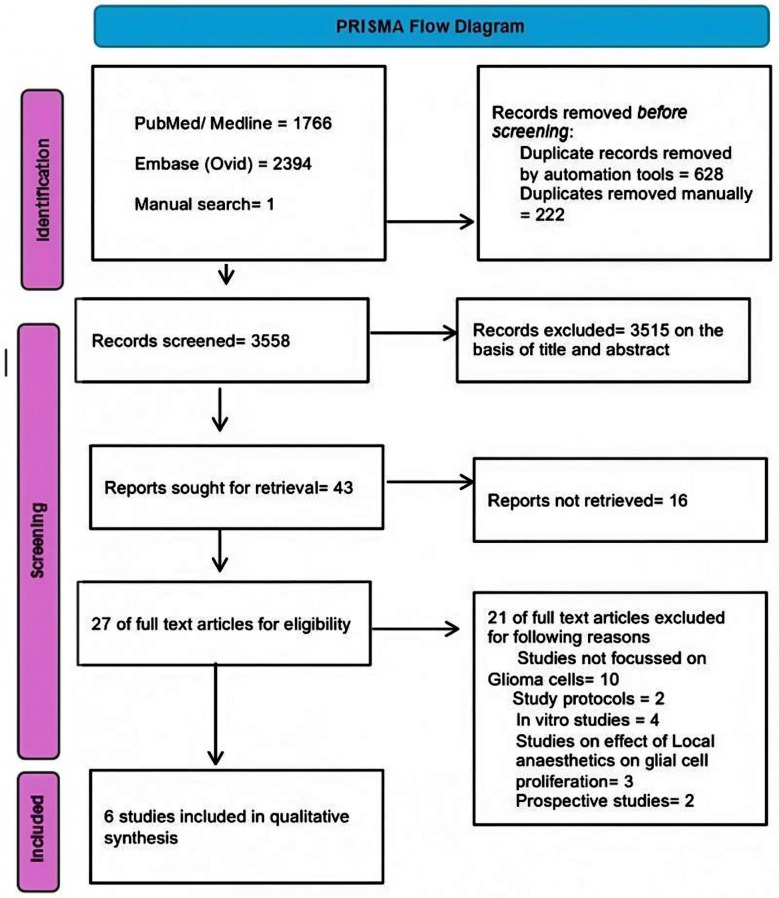
Preferred Reporting Items for Systematic Reviews and Meta-Analysis (PRISMA) flow chart for included studies.

Our initial search across databases retrieved 3779 articles. We removed 222 duplicates and excluded 2037 articles after reviewing titles, then eliminated another 1478 based on abstract screening. Following a full-text evaluation, 37 additional articles were excluded, leaving five articles for analysis. A manual check of the reference lists for these articles identified one more study, resulting in a total of six articles. The entire screening process is illustrated in the PRISMA flow diagram (Fig.1).

### Study characteristics:

A total of six retrospective cohort studies conducted between 2017 and 2021 were reviewed, with study sites located in the USA, Germany & China, two each.^18^ The summary of participant characteristics, key results, and trial locations is provided in Table-II (given in attached file). All studies included both male and female participants, predominantly aged over 18 years, except for one study that enrolled patients as young as 16.^19^ One multi-center study used combined data to compare the effects of minimal sedation versus awake craniotomy, and its secondary analysis contrasted total intravenous anaesthesia (TIVA) with volatile anesthetics.^20^ Four studies focused on comparing inhalational anaesthesia with TIVA as the primary outcome, and one study examined the effect of inhalational anaesthesia versus combination anesthesia. Only one study had a sample size of 891, while all other studies had much smaller sample sizes.^20^

**Table-II:** Summary of Included studies

Study	Sample size	Age group	KPS	Inclusion criteria	Time to FU	Post-surgical Chemotherapy
Grau et al.,2020^17^	150	>18	70-100	age, gender, KPS, choice & duration of anaesthesia, dose of intra-op opioids, postop FU	18 months	Yes
Cata et al., 2017^18^	378	>18	NA	age, gender, BMI, ASA status, anaesthesia duration, adjuvant radio-chemotherapy	3 and 5 years	Yes
Dong et al., 2019^19^	294	>16	70-80	duration of anaesthesia, use of opioids, intra-op hemodynamic, postop FU	12 months	Yes
Chowdhury et al., 2021^20^	891	>18	>70	age, gender, awake craniotomy vs GA, Duration of surgery/anaesthesia, periop/postop complications, postop FU	Couldn’t determine	Yes
Huang et al., 2021^21^	103	>20	<70 or 80-100	age, KPS, ASA score, type of anaesthesia, duration of surgery/anaesthesia, dosage of opioids, peri/postop complications, postop FU	36 months	Yes
Schomoch et al., 2021^22^	471	>18	mean 82	age, gender, primary tumour, ASA score, radio-chemotherapy, KPS, extent of resection, postop FU	24 months	Yes

postop FU (adio-chemo, time to progression, recurrence, death).

### Outcome comparison:

Only Huang et al. associated propofol with increased PFS and OS^21^, rest of the studies showing no effect while Dong et al. suggested that sevoflurane may reduce OS in patients with KPS <80.^19^ Overall, the type of general anaesthesia drugs did not affect five-year PFS rate. These findings are summarized in Table-III. The critical analysis of these studies showed that 83% of the studies included in review showed no significant difference on primary or secondary outcome of patients.

**Table-III:** Outcomes of included studies

Study	Choice of anaesthesia	Primary outcomes	Secondary outcomes
Grau et al.,2020^17^	INH vs TIVA	No significant difference in PFS and OS among volatile vs propofol	MGMT promotor methylation status, gross total resection & age <70 affected PFS
Cata et al., 2017^18^	INH(isoflurane vs desflurane) vs combination	Volatile anaesthetics do not have negative impact on PFS & OS	Type of GA techniques has no impact on 5 year PFS rates
Dong et al., 2019^19^	INH vs TIVA	Sevoflurane & propofol didn’t impact PFS & OS,sevoflurane decreased OS in pts. with KPS <80	Chemotherapy improved OS, longer duration of anaesthesia reduces PFS and OS
Chowdhury et al., 2021^20^	Awake vs GA	No PFS difference of awake and GA anaesthesia, awake craniotomy and OS had a positive association but not statistically significant after controlling variables	Within GA group, no impact on OS between TIVA and INH anaesthetics
Huang et al., 2021^21^	INH(desflurane) vs TIVA	Propofol group associated with increased PFS and OS	
Schomoch et al., 2021^22^	INH vs TIVA	IONM had no impact on OS	IONM had no impact on OS

INH: Inhalational, GA: General anaesthesia, TIVA: Total IV anaesthesia.

## DISCUSSION

Analyzing progression-free survival (PFS) & overall survival (OS) due to choice of anaesthetic agent in glioma resection, five out of six studies reported no significant difference in progression-free survival (PFS) between patients receiving volatile anaesthetics (isoflurane, desflurane, or sevoflurane) and those administered total intravenous anaesthetics or a combination of both. The study by Huang et al.^21^ found that overall and cancer-related mortality were significantly lower in patients receiving propofol (72%) compared to those receiving desflurane (90%), with a corresponding improvement in overall survival. Though limited by small sample size (103 patients), its findings are particularly notable given that the mean Karnofsky Performance Status (KPS) scores were similar between the groups (88 for propofol and 87 for the inhalational group), indicating a reliable comparative baseline.^21^ The only striking finding in other studies was increased risk of death in sevoflurane group compared to propofol group in patients who had Karnofsky performance status <80 noted by Dong et al. The hazard ratio was 1.66 with 95% confidence interval.^19^ Another difference noted in these articles was that Schmoch et al. included only patients with IDH-1 mutation,^22^ and Grau et al. excluded them owing to different prognosis of secondary GBM.^17^ But there has been no difference in the outcome of these two in terms of PFS and OS.^17^,^22^

However, the limited number of studies on the topic makes it difficult to draw definitive conclusions, and the methods employed across these studies vary significantly, which was already observed in a narrative review.^23^ The effect of propofol also remains controversial in experimental studies. It has been found to promote the tumour initiation of glioma cells in mice.^13^ Similar results were found in a molecular study where propofol inhibited metabolic pathways, upregulated immune checkpoint molecules and promoted immune escape of glioma during surgery whilst suppressing SERPINI-1, overall favouring the progression of Gliomas.^12^ In addition, these findings are inconsistent with another study highlighting the ability of propofol as an anti-cancer agent which can inhibit the crosstalk of glioma cells with the tumour microenvironment and does not have any cytotoxic effects on mature neural cells.^24^ Furthermore, the findings related to volatile anaesthetics are more mixed. Isoflurane, a commonly used volatile anaesthetic, has been shown to increase the invasion and growth of glioblastoma multiforme (GBM) stem cells, potentially through mechanisms involving VEGF expression.^25^ Furthermore, some studies suggest that volatile anaesthetics are linked to higher mortality in patients undergoing cancer surgery.^26^ In contrast, other studies highlight potential benefits of inhalational agents, such as Sevoflurane, in improving recovery and prognosis in glioma patients.^11^,^12^ Interestingly, the carcinogenic effects of isoflurane observed in vitro may not apply to the perioperative environment, which is hypoxic and differs significantly from the controlled conditions of in vitro experiments.^6^

Consequently, the clinical implications of these findings are particularly relevant for anaesthetic choices in glioma surgeries. For example, a narrative review aligns with the findings, suggesting that anaesthetics like propofol, which suppress neuroendocrine responses through the HPA axis and SNS, may reduce immunosuppression and lower the risk of cancer recurrence compared to volatile anaesthetics and opioids, which may have negative effects on immune function.^4^ Propofol has also been found to increase levels of IL-10, an inflammatory cytokine, suggesting anti-inflammatory effects.^7^ However, these effects do not necessarily improve the oncological outcomes. While the research into the effects of anaesthetics on glioma progression and survival is still emerging, the current studies are inconclusive, mostly because of the variability in study design, sample size, and the types of anaesthetics examined. Additionally, factors such as local anaesthetics (e.g., scalp blocks and lidocaine), which have shown promise in improving progression-free survival in glioma surgeries, were not consistently considered in the studies reviewed.^27^,^8^ Therefore, future research must focus on large-scale, well-controlled trials to precisely determine the clinical implications of anaesthetic selection in glioma treatment. Unravelling the influence of various anaesthetic agents on tumour progression, immune modulation, and patient outcomes could pave the way for optimized perioperative strategies. This, in turn, may enhance long-term survival, reduce recurrence rates, and improve overall quality of life for glioma patients undergoing surgical intervention.

### Strengths and Limitations:

Strengths for this review entail thorough electronic database search, supplemented by manual reference screening, ensured a comprehensive collection of relevant literature, independent data extraction by minimum two reviewers. No restriction on race, geography, sex, age, ethnicity, or language were applied. This review is limited by the small number of available studies although we do acknowledge that electronic search was conducted on to two databases only. Also, their retrospective nature introduces the possibility of selection bias, as patient inclusion criteria and data availability may have influenced the results. Additionally, sample sizes varied, with most studies involving fewer than 500 participants. The research timeline is also limited, with all available studies conducted within five years and the most recent one published four years ago which restricts sufficient data to guide clinical guidelines. The lack of ongoing investigations in this area is reflected in the fact that only two RCTs are currently in progress. Lastly, due to significant differences between studies, a meta-analysis was not feasible, limiting the ability to draw definitive conclusions.

## CONCLUSION

Five out of six studies found no relevance of tumour progression with anaesthetic agents. The literature suggesting potential tumour-modulating effects of these drugs is far more convincing to warrant further studies. . Similar associations have been reported in other cancer types, indicating a possible role of anaesthesia in tumour progression outcomes. Well-designed Randomized controlled trials are crucial to clarify if anaesthetic agents affect long-term survival and oncological outcomes in glioma patients.

### Recommendations

We recommend designing rigorous prospective randomized controlled trials specifically looking at type of anaesthetic agent & its long-term outcome in glioma resection, to have larger cohorts that could play a potentially benefitting role to improve long term outcomes, dictate clinical decisions & guidelines.

### Author`s Contribution:

**AM:** Conceived the idea, collected and analyzed data, interpreted results and drafted manuscript.

**SA, AK and MN:** Collected and analyzed the data, interpreted the results and drafted the manuscript

**HMQ and SSHS:** Supervision, Design of the study. Critical Review.

All the authors have read and approved the final manuscript and are responsible and accountable for the accuracy and integrity of the work.
